# Impact of systemic dexamethasone administration on oral mucositis induced by anthracycline-containing regimens in breast cancer treatment

**DOI:** 10.1038/s41598-022-16935-4

**Published:** 2022-07-22

**Authors:** Yoshitaka Saito, Yoh Takekuma, Takashi Takeshita, Tomohiro Oshino, Mitsuru Sugawara

**Affiliations:** 1 Department of Pharmacy, Hokkaido University Hospital, Kita 14-jo, Nishi 5-chome, Kita-ku, Sapporo, 060-8648 Japan; 2 Department of Breast Surgery, Hokkaido University Hospital, Kita 14-jo, Nishi 5-chome, Kita-ku, Sapporo, 060-8648 Japan; 3 Laboratory of Pharmacokinetics, Faculty of Pharmaceutical Sciences, Hokkaido University, Kita 12-jo, Nishi 6-chome, Kita-ku, Sapporo, 060-0812 Japan

**Keywords:** Breast cancer, Risk factors

## Abstract

Oral mucositis (OM) is one of the most common complications associated with chemotherapy. Here, we evaluated whether systemic dexamethasone (DEX) dosage in prophylactic antiemetics affected the incidence of OM in anthracycline-containing regimens. Patients receiving anthracycline-containing regimens for breast cancer were divided into high- and low-DEX dose groups and retrospectively evaluated. The incidence of all-grade OM in the first cycle in the high- and low-dose groups was 27.3% and 53.5%, respectively, and was significantly lowered by increasing the DEX dose (*P* < 0.01); thus, the study met its primary endpoint. The result in all treatment cycles was also significant (*P* = 0.02). In contrast, the incidence of dysgeusia was similar between the high- and low-dose groups in the first and all cycles (13.6% and 16.3% in the first cycle [*P* = 0.79] and 27.3% and 34.9% in all cycles [*P* = 0.42], respectively). Multivariate analysis revealed that low DEX dosage was an independent risk factor for all-grade OM development. In conclusion, our study suggests that DEX attenuates OM in anthracycline-containing regimens for breast cancer treatment in a dose-dependent manner. Further evaluation of OM prophylaxis, including DEX administration, is required for better control.

## Introduction

Oral mucositis (OM) is one of the most common complications of chemotherapy, radiotherapy, chemoradiotherapy, and hematopoietic stem cell transplantation^1^. It is a painful inflammatory, often ulcerative condition, and is associated with reduced food and water intake, need for parenteral nutrition, and systemic analgesic administration in some cases, leading to a decrease in quality of life (QOL) and treatment dosage. Severe symptoms also increase the risk of systemic infections, inpatient hospitalization duration, and 100-day mortality owing to the disrupted oral mucosal barrier^2–4^.

Anthracyclines are key agents used in conjunction with cyclophosphamide for breast cancer treatment. Epirubicin (90 mg/m^2^) + cyclophosphamide (600 mg/m^2^) (EC), every 3 weeks; epirubicin (100 mg/m^2^) + cyclophosphamide (500 mg/m^2^) + 5-fluorouracil (500 mg/m^2^) (FEC), every 3 weeks; and dose-dense doxorubicin (60 mg/m^2^) + cyclophosphamide (600 mg/m^2^) (AC), every 2 weeks, are frequently used in perioperative breast cancer treatment, and approximately 40–50% of patients experience OM^5–8^.

The current understanding of chemotherapy-induced OM pathophysiology is (1) initiation of oral mucosal damage, (2) primary damage from reactive oxygen species generation, (3) damage amplification due to the host inflammation response, and (4) mucosal ulceration as a result of epithelial apoptosis and necrosis, and ultimately followed by (5) healing^9–11^. Inflammation is considered as an important tissue reaction in chemotherapy- and radiotherapy-induced OM^12,13^. Proinflammatory cytokines such as tumor necrosis factor α and interleukin 1β play pivotal roles in the pathogenesis of OM^14–16^. In addition, the levels of nuclear factor kappa-B and cyclooxygenase-2 in the oral mucosa significantly increase following cytotoxic chemotherapy^17^. In contrast, benzydamine mouthwash is a singular recommended preventive anti-inflammatory OM medication in specific patient populations^12^. Dexamethasone (DEX) is a long-acting corticosteroid recommended for chemotherapy-induced nausea and vomiting (CINV) prevention^18^. At Hokkaido University Hospital, antiemetic regimens for anthracycline-containing treatment include DEX, palonosetron (a serotonin receptor antagonist), and aprepitant (a neurokinin-1 receptor antagonist). However, the DEX dosage used was as follows: 6.6 mg infusion on day 1 and 4 mg orally on days 2–4. This DEX dosage was altered to 9.9 mg infusion on day 1 and 8 mg orally on days 2–4, in accordance with the national guidelines^19^. In this study, we evaluated whether DEX dosage alteration affected OM incidence and severity in anthracycline-containing regimens in a real-world setting.

## Results

### Patient characteristics

**Figure 1:**
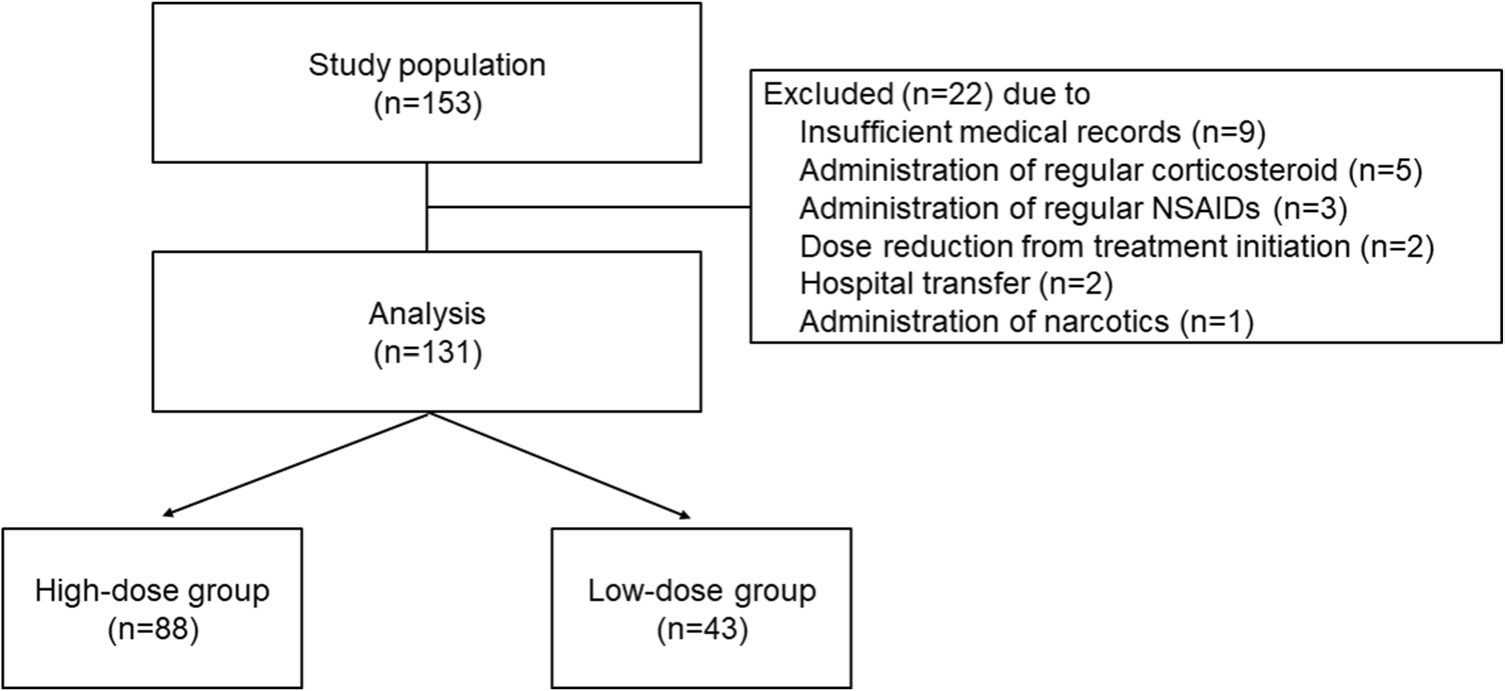
Design of this study. *NSAIDs* non-steroidal anti-inflammatory drugs.

One hundred and thirty-one patients were enrolled according to the eligibility criteria for this study (Fig. 1). Baseline patient characteristics are shown in Table 1. There were no significant differences between the two groups in the Eastern Cooperative Oncology Group performance status (ECOG PS), staging, presence of lymph node metastases, hormonal receptor expression, prior treatment existence, menopause, body surface area (BSA), liver dysfunction (grade 1 or higher aspartate aminotransferase, alanine aminotransferase, and total bilirubin level elevation), renal dysfunction (grade 1 or higher serum creatinine level elevation), serum albumin level, regular alcohol intake (≥ 5 days per week), smoking history, and regular administration of antacids. Regular oral care by dentists was conducted in 70% of the patients, and its implementation rate did not differ between the groups. Baseline oral condition was also not different, although 31.8% of the high-dose and 25.6% of the low-dose patients were not evaluated. In contrast, patients in the high-dose group were significantly older, received more EC or dose-dense AC than FEC and more pegfilgrastim administration, and showed lower human epidermal growth factor receptor 2 (HER2) overexpression than those in the low-dose group.

**Table 1:** Patient characteristics.

	High-dose group (n = 88)	Low-dose group (n = 43)	*P* value
Age (median, range)	55 (26–73)	50 (32–66)	0.01*
**Performance status**			
0–1	88	43	1.00
**Staging**			
I–III	83	40	
IV/Recurrence	5	3	0.72
Presence of Lymph node metastases	45	19	0.46
**Hormonal receptors**			
ER, PR-positive or both	47	17	0.14
HER2 overexpression	20	19	0.02*
Prior treatment existence	10	3	0.54
Menopause	55	22	0.26
BSA (m^2^) (median, range)	1.56 (1.33–2.02)	1.55 (1.34–1.92)	0.71
Liver dysfunction	31	16	0.85
Renal dysfunction	11	2	0.22
Serum albumin (g/dL) (median, range)	4.2 (3.5–4.8)	4.2 (3.8–4.9)	0.08
Alcohol intake (≥ 5 days in a week)	17	9	0.82
**Smoking history (former or current)**	47	21	0.71
Current smoker	13	3	0.26
Implementation of dental oral care	60	32	0.54
**Oral condition assessment by dentist**			
No problem	31	19	
Need for any dental treatment	29	13	0.52
Regular antacid administration	5	0	0.17
Pegfilgrastim administration	38	2	< 0.01**
**Treatment regimen**			
AC or EC	74	6	
FEC	14	37	< 0.01**

Liver dysfunction: grade 1 or higher aspartate aminotransferase, alanine aminotransferase, and total bilirubin levels.

Renal dysfunction: grade 1 or higher serum creatinine elevation.

Antacids include proton pump inhibitors and histamine type 2 receptor antagonists.

*ER* estrogen receptor, *PR* progesterone receptor, *HER2* human epidermal growth factor receptor 2, *BSA* body surface area, *AC* doxorubicin (60 mg/m^2^) + cyclophosphamide (600 mg/m^2^), *EC* epirubicin (90 mg/m^2^) + cyclophosphamide (600 mg/m^2^), *FEC* epirubicin (100 mg/m^2^) + cyclophosphamide (500 mg/m^2^) + 5-fluorouracil (500 mg/m^2^).

*P < 0.05.

**P < 0.01.

### Comparison of the OM and dysgeusia incidence

**Figure 2:**
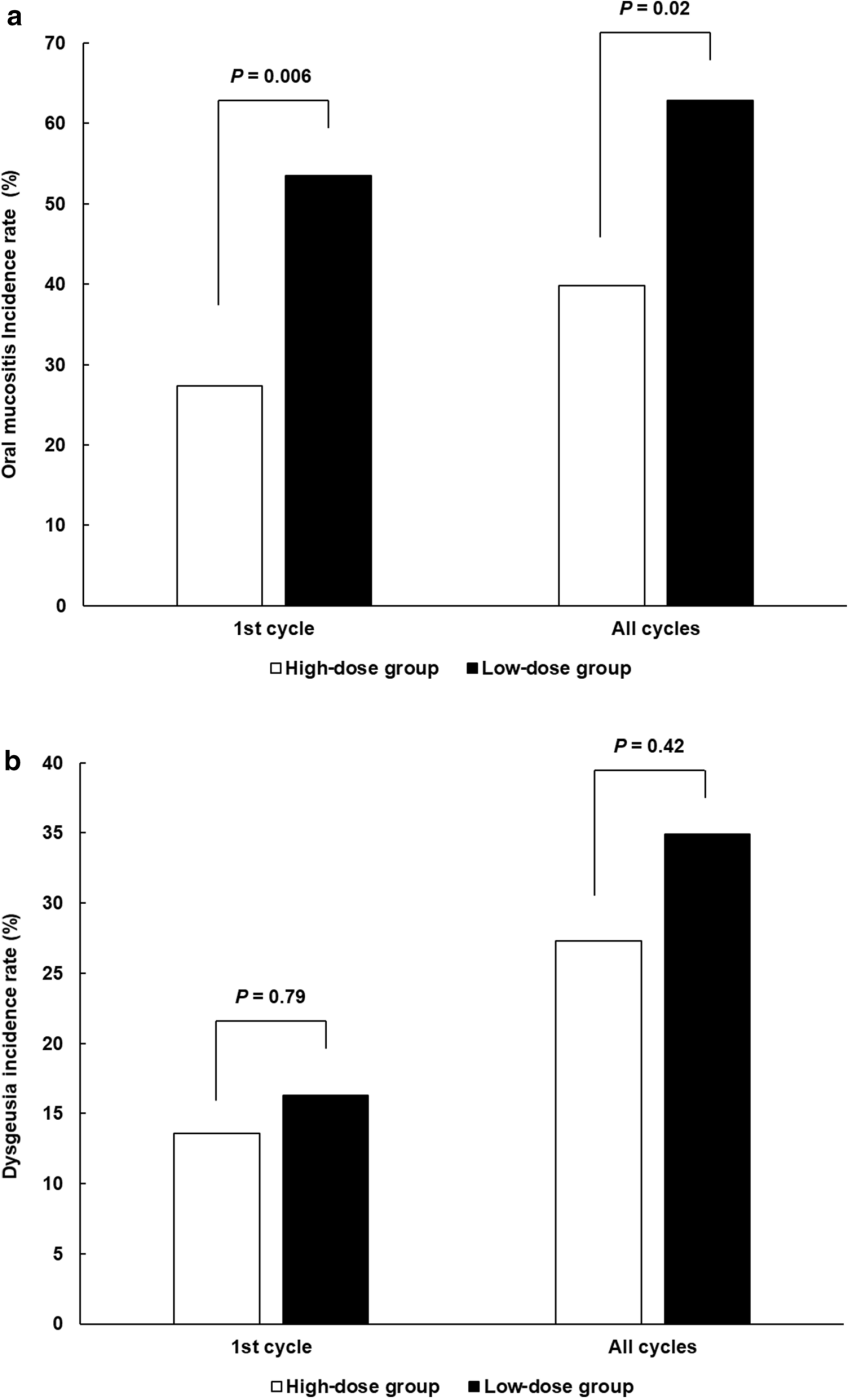
Comparison of all-grade (**A**) OM and (**B**) dysgeusia incidence between high- and low-DEX-dose groups in the first cycle and all treatment cycles.

Figure 2 shows a comparison of OM and dysgeusia incidence between the two groups. The difference in the rate of all-grade OM incidence in the first cycle between the two groups was defined as the primary endpoint of this study: the rate was 27.3% in the high-dose group and 53.5% in the low-dose group and was significantly lowered by DEX dose increase (*P* < 0.01); the rate in all treatment cycles was 39.8% in the high-dose group and 62.8% in the low-dose group, showing significant difference (*P* = 0.02, Fig. 2A). The incidence of grade 2 OM was not statistically different between the high- and low-dose groups in the first and all cycles (2.3% and 9.3% in the first cycle [*P* = 0.09] and 5.7% and 9.3% in all cycles [*P* = 0.47], respectively). None of the patients experienced any grade 3/4 symptoms. In contrast, dysgeusia similarly appeared between the high- and low-dose groups in the first and all cycles (13.6% and 16.3% in the first cycle [*P* = 0.79] and 27.3% and 34.9% in all cycles [*P* = 0.42], respectively) (Fig. 2B).

### Assessment of the risk factors for OM incidence

Multivariate analysis was performed to identify independent risk factors for all-grade OM incidence in the first cycle of treatment according to previous reports^20–24^. As a result, a lower DEX dosage was revealed to be an independent risk factor for OM development (Table 2).

**Table 2:** Univariate and multivariate analyses of the risk factors associated with the incidence of all-grade oral mucositis in the first cycle.

	Univariate analysis		Multivariate analysis	
	Odds ratio (95% CI)	*P* value	Odds ratio (95% CI)	*P* value
**Age (years)**				
< 55/≥ 55	0.84 (0.33–2.13)	0.72	Excluded	–
**BSA (m**^**2**^**)**				
> 1.5/≤ 1.5	0.98 (0.47–2.06)	0.96	Excluded	–
**Staging**				
I–III/IV or recurrence	0.31 (0.07–1.37)	0.12	0.34 (0.07–1.59)	0.17
**Prior treatment**				
Yes/no	1.13 (0.35–3.68)	0.84	Excluded	–
**Hormonal receptors**				
ER-, PR-positive or both/Negative	0.67 (0.33–1.38)	0.28	Excluded	–
**HER2 overexpression**				
Positive/negative	1.86 (0.86–4.01)	0.11	1.62 (0.71–3.69)	0.25
**Alcohol intake (≥ 5 days in a week)**				
Yes/no	1.15 (0.47–2.79)	0.76	Excluded	–
**Smoking history**				
Current/former or never	1.46 (0.50–4.21)	0.49	Excluded	–
**Menopause**				
Yes/no	0.70 (0.34–1.44)	0.33	Excluded	–
**Hypoalbuminemia**				
Present/absent	0.80 (0.36–1.78)	0.58	Excluded	–
**Liver dysfunction**				
Present/absent	1.02 (0.48–2.15)	0.96	Excluded	–
**Renal dysfunction**				
Present/absent	0.78 (0.23–2.67)	0.69	Excluded	–
**Implementation of dental oral care**				
Yes/no	1.17 (0.53–2.58)	0.69	Excluded	–
**Regular administration of antacids**				
Yes/no	0.43 (0.05–4.01)	0.46	Excluded	–
**Administration of pegfilgrastim**				
Yes/no	0.40 (0.17–0.95)	0.04*	0.63 (0.24–1.63)	0.34
**Dexamethasone dosage**				
Low/high	3.07 (1.43–6.56)	0.004**	2.38 (1.01–5.60)	0.048*

Liver dysfunction: grade 1 or higher aspartate aminotransferase, alanine aminotransferase, and total bilirubin levels.

Renal dysfunction: grade 1 or higher serum creatinine level elevation.

Antacids include proton pump inhibitors and histamine type 2 receptor antagonists.

Cutoff of the serum albumin levels is 4.1 g/dL at our facility.

*CI* confidence interval, *BSA* body surface area, *ER* estrogen receptor, *PR* progesterone receptor, *HER2* human epidermal growth factor receptor 2.

*P < 0.05.

**P < 0.01.

## Discussion

OM is a problematic chemotherapy-induced adverse effect. Its incidence is associated with pain, difficulty in eating and swallowing, and bacteremia, resulting in treatment interference, which can lead to a reduction in the chemotherapeutic dosage and QOL of patients^1^. There have been no reports evaluating the preventive efficacy of systemic corticosteroids against OM, and evidence of OM prophylaxis is limited. Consequently, we aimed to assess the OM preventive effect of systemic DEX in a real-world setting.

As a result, DEX administration based on the HEC preventive strategy significantly reduced the incidence of all-grade OM compared to a lower DEX dosage. Moreover, its administration tended to decrease ≥ grade 2 symptom development, but without statistical significance. We have previously reported that prolonged DEX administration attenuates taxane-associated acute pain syndrome, the main cause of which is inflammation, owing to the strong anti-inflammatory effect of DEX^25,26^. Therefore, we consider that DEX prevents OM incidence in a dose-dependent manner by reducing chemotherapy-induced inflammation in the oral mucosa. Anti-inflammatory agents can be an OM preventive strategy considering their mechanism; however, benzydamine mouthwash is a singular OM preventive anti-inflammatory medication recommended by the Multinational Association of Supportive Care in Cancer and International Society for Oral Oncology (MASCC/ISOO) guidelines^1^. The results of this study showed new findings regarding chemotherapy-induced OM prevention using anti-inflammatory agents. Steroid sparing is one of the most considerable attentions in CINV management^27–29^ as corticosteroid administration induces increased susceptibility to infection, especially to pneumocystis pneumonia (PCP), insomnia, reduced bone mineral densities, and blood sugar level elevation^30–32^. However, it is unknown whether this strategy affects other adverse effects that have not been mentioned in previous studies^27–29^. If a patient experiences OM with an increase in the burden of anthracycline-containing treatment with steroid sparing, DEX administration according to the HEC guidelines can be used as a management strategy, particularly in perioperative breast cancer chemotherapy, as dose intensity reduction increases the annual odds of recurrence^33–35^. However, further evaluation of the most suitable DEX dosage and duration for OM prophylaxis is required.

Treatment and patient factors affect OM risk^36^. Treatment factors include the type, dose, and schedule of systemic chemotherapeutic drugs; radiation dose and field; and concomitant use of chemotherapy and radiation. In addition, the risk of OM increases as the intensity of therapy increases^37^. Patient-related risk factors are complicated and poorly defined^22^. Age, malnutrition, male sex, pre-existing medical conditions, alterations in salivary production and composition, poor oral health, tobacco smoking, low dental checkup frequency, and mucosal trauma have been reported to influence the risk^20–22,24^. In this study, we evaluated the risk factors based on these reports and found that lower DEX administration is the only independent risk factor for all-grade OM incidence.

In the MASCC/ISOO systematic review, the panel is of the opinion that dental evaluation and treatment prior to cancer therapy is desirable to reduce the risk of local and systemic infections from odontogenic sources, although there is insufficient evidence to support^9^. In this study, dental professional oral care was not associated with OM incidence in the first cycle. However, approximately 30% of the patients in each group did not receive professional oral care, and oral treatment timing was different for each patient. Even though the patients received baseline assessment, our evaluation might have been insufficient. Therefore, further evaluation using an integrated intervention protocol is required.

This study had some limitations regarding the evaluation of the impact of DEX dosage on the incidence of OM in anthracycline-containing treatments. First, this study was retrospective and included a relatively small patient population from a single institution. Second, we evaluated the OM incidence between different DEX doses; therefore, it is necessary to compare patients with and without DEX administration, particularly on days 2–4. Third, we did not fully circumstantially assess the implementation of oral rinse, although almost all patients performed correctly, and its efficacy remains unclear. Finally, patients in the high-dose group were significantly older than those in the low-dose group, and older age has previously been reported as an independent OM risk factor; however, our study did not confirm this finding. In addition, pegfilgrastim was administered more frequently in the high-dose group than in the low-dose group. A previous report suggested that granulocyte colony-stimulating factor can effectively treat and prevent doxorubicin-induced OM^38^, although this study's co-administration was not associated with OM incidence. Furthermore, treatment regimens were significantly different between the groups, although the impact on the results was likely low, as the reported OM incidences in AC/EC and FEC treatments were not different^5–8^; however, it might have affected the results. Consequently, evaluation of well-balanced patients will enable the derivation of better outcomes.

In conclusion, our study suggests that DEX reduces the incidence of OM in anthracycline-containing regimens for breast cancer treatment in a dose-dependent manner. Further evaluation of OM prophylaxis consisting of medication and other methods such as dental care or cryotherapy and the DEX administration method will provide good OM-controlling treatment, leading to less onerous and effective chemotherapy.

## Methods

### Patients

Female patients with breast cancer who received anthracycline-containing regimens were retrospectively evaluated. The administered regimens were EC, FEC, and dose-dense AC. All patients met the following baseline criteria: (1) age ≥ 20 years, (2) 0–2 ECOG-PS, and (3) sufficient renal and liver function for chemotherapy induction. Patients who were previously administered anthracyclines, regularly dosed corticosteroids, non-steroidal anti-inflammatory drugs, narcotics, or reduced chemotherapy from initiation; diagnosed with OM at baseline; transferred to another hospital during the chemotherapy; and without sufficient information were excluded. The patients were divided into two groups: the high-dose group, which included patients administered 9.9 mg DEX infusion on day 1 and 8 mg orally on days 2–4 between April 2017 and September 2021, and the low-dose group, which included patients administered DEX 6.6 mg infusion on day 1 and 4 mg orally on days 2–4 between February 2016 and January 2018.

The present study was approved by the Ethical Review Board for Life Science and Medical Research of the Hokkaido University Hospital (approval number: 021-0179) and was performed in accordance with the Declaration of Helsinki. Owing to the retrospective nature of the study, informed consent from the subjects was waived by the committee.

### Treatment methods

The treatment schedule for EC, FEC, and dose-dense AC has been previously described. Antiemetic therapy consisting of palonosetron 0.75 mg on day 1 and aprepitant (125 mg on day 1 and 80 mg on days 2 and 3) was administered to all the participants. DEX was administered as previously described. All patients were prescribed a sodium gualenate hydrate gargle and strongly recommended rinsing three times a day. Steroid oral ointment and gargle, lidocaine gargle, and systemic analgesics were administered for OM treatment at the physician’s discretion.

### Evaluation of OM and dysgeusia

All the required information was obtained from the patients’ medical records. We recommend that all patients maintain their daily diaries. We assessed oral adverse effects by referring to the diary and patient complaints in accordance with the Common Terminology Criteria for Adverse Events, version 5.0, provided by physicians or pharmacists. In this study, the primary endpoint was the comparison of all-grade OM incidence in the first cycle between the groups. Secondary endpoints included the evaluation of OM incidence in all cycles and dysgeusia incidence, and risk factor analysis for all-grade OM incidence in the first cycle.

### Statistical analysis

We hypothesized that the all-grade OM incidence would be 25–30% in the high-dose group and 55% in the low-dose group, with a patient ratio of 2:1 based on previous reports and our clinical experiences^5–8^. To achieve 80% power with an alpha error of 5%, the required sample size was 70–102 participants in the high-dose group and 35–51 participants in the low-dose group. We included 88 and 43 patients in the high- and low-dose groups, respectively.

The differences in baseline patient clinical characteristics between the high- and low-dose groups were assessed using Fisher’s exact probability test for categorical outcome variables and Mann–Whitney U test for continuous parameters. The incidences of OM and dysgeusia were compared using Fisher’s exact probability test. Logistic analyses were performed to identify independent all-grade OM risk factor(s) in the first cycle of treatment. Potential baseline risk factors included age, BSA, staging, prior treatment, hormonal receptor expression, HER2 overexpression, regular alcohol intake, smoking history, menopause, hypoalbuminemia, liver dysfunction, renal dysfunction, dental oral care implementation, regular administration of antacids such as proton pump inhibitors or histamine type 2 receptor antagonists, pegfilgrastim co-administration, and DEX dosage, according to previous reports^20–24^. Variables that had potential associations with OM incidence in the first cycle, as suggested by the univariate logistic regression analysis (*P* < 0.20), were considered when building the multivariable model. All analyses were performed using JMP statistical software (version 14.0; SAS Institute Japan, Tokyo, Japan). Differences were considered statistically significant when the *P* value was less than 0.05.

### Ethics approval and consent to participate

All procedures performed in this study were carried out in accordance with the ethical standards of the institutional and/or national research committee and the 1964 Helsinki Declaration and its later amendments or comparable ethical standards. The study was approved by the Ethical Review Board for Life Science and Medical Research of Hokkaido University Hospital (approval number: 021-0179). The requirement for formal consent for this type of study was waived by the committee.

## Data Availability

The datasets used and/or analyzed in the current study are available from the corresponding author upon reasonable request.
